# Computational Systems Biology Approach for the Study of Rheumatoid Arthritis: From a Molecular Map to a Dynamical Model

**DOI:** 10.18547/gcb.2018.vol4.iss1.e100050

**Published:** 2017-12-06

**Authors:** Vidisha Singh, Marek Ostaszewski, George D. Kalliolias, Gilles Chiocchia, Robert Olaso, Elisabeth Petit-Teixeira, Tomáš Helikar, Anna Niarakis

**Affiliations:** 1 GenHotel EA3886, Univ Evry, Université Paris-Saclay, 91025, Evry, France; 2 Luxembourg Centre for Systems Biomedicine, Université du Luxembourg, Esch-sur-Alzette, Luxembourg; 3 Arthritis and Tissue Degeneration Program, Hospital for Special Surgery, New York, USA; Department of Medicine, Weill Cornell Medical College, New York City, USA; 4 Faculty of Health Sciences Simone Veil, INSERM U1173, University of Versailles Saint-Quentin-en-Yvelines, Montigny-le-Bretonneux, France; 5 Centre National de Recherche en Génomique Humaine (CNRGH), CEA, Evry, France; 6 Department of Biochemistry, University of Nebraska-Lincoln, Lincoln, NE, USA

**Keywords:** Complex human disease, Rheumatoid arthritis, Computational systems biology, Interactive molecular map, Signaling network, Dynamical modelling

## Abstract

In this work we present a systematic effort to summarize current biological pathway knowledge concerning Rheumatoid Arthritis (RA). We are constructing a detailed molecular map based on exhaustive literature scanning, strict curation criteria, re-evaluation of previously published attempts and most importantly experts’ advice. The RA map will be web-published in the coming months in the form of an interactive map, using the MINERVA platform, allowing for easy access, navigation and search of all molecular pathways implicated in RA, serving thus, as an on line knowledgebase for the disease. Moreover the map could be used as a template for Omics data visualization offering a first insight about the pathways affected in different experimental datasets. The second goal of the project is a dynamical study focused on synovial fibroblasts’ behavior under different initial conditions specific to RA, as recent studies have shown that synovial fibroblasts play a crucial role in driving the persistent, destructive characteristics of the disease. Leaning on the RA knowledgebase and using the web platform Cell Collective, we are currently building a Boolean large scale dynamical model for the study of RA fibroblasts’ activation.

Protein-protein interactions are a major driving force behind most biological processes. They play a pivotal role in intra- and extra-cellular functions, and especially in the propagation of signals and cellular regulation. Signal transduction is a fundamental process for the communication of the cell with its environment, comprising several interacting receptors, proteins, enzymes, second messengers and transcription factors. Disruption and dysregulation of these complex molecular and signaling networks can lead to disease. Therefore, the mapping and accurate representation of pathways implicated is a primary but essential step for elucidating the mechanisms underlying disease pathogenesis.

The release of various molecular maps dealing with obesity [1], gastrin and cholecystokinin receptors signaling [2], FceRI receptor signaling in allergy [3], MAPKs [4], mTOR signaling [5] to name a few, corroborates the fact that pathway assembly in the form of network is gaining ground in systems biology. As more scientists invest time and effort to construct large molecular networks, there is an increasing need for practical guidelines and a standardized framework. Toward this direction, initiatives have emerge, such as The Cancer Cell Map Initiative [6], the Atlas of Cancer Signaling Networks (http://acsn.curie.fr) concerning cancer, and the Disease Maps Project (http://disease-maps.org), an open, large-scale community effort that consists of a network of groups working together for developing best practices, standards and tools in order to better represent disease mechanisms.

However, as all living systems are dynamic in nature, static representations of molecular networks can provide useful but relatively limited understanding. A dynamical study can reveal information about the system’s behavior under different conditions by *in silico* simulations, perturbations, hypotheses testing and predictions. Quantitative kinetic modelling approaches using differential or stochastic equations can provide a detailed analysis of a network’s dynamics, but the large number of parameters required make them less appropriate for large scale signaling networks. In order to address the lack of kinetic data, discrete logical modelling can be used as an alternative way to study the system’s qualitative dynamic behavior [7, 8].

In this work we present a systematic effort to summarize current biological pathway knowledge concerning Rheumatoid Arthritis (RA), a multifactorial autoimmune disease that causes chronic inflammation of the synovial joints with an etiology that still remains unclear. With the use of the software CellDesigner [9], we are constructing a detailed molecular map based on exhaustive literature scanning, strict curation criteria, re-evaluation of previously published attempts [10] and most importantly experts’ advice (Figure 1).

In 2010 Wu *et al.* published a detailed molecular map concerning rheumatoid arthritis using the software CellDesigner. We decided to use this map as a basis, and expand. The map has been updated with information published after 2010 by exhaustive manual curation and the help of data mining tools. Only experimentally validated interactions in at least two peer reviewed scientific publications are kept. Due to the fact that the initial map was based on high throughput gene expression data from 28 studies and interactions inferred from KEGG database, all nodes and interactions are re-evaluated carefully in an effort to limit false positives. When validation with small scale experiments is not possible, we keep nodes that appear in at least two different high throughput studies. Detailed annotation including PubMED IDs and HUGO names is also added in the MIRIAM section of the CellDesigner file. As far as context representation and overall structure of the map, expert’s advice has been taken into account along with an effort to comply with SBGN standards.

The RA map will be web-published in the coming months (a full length manuscript is under preparation) in the form of an interactive map, using the platform MINERVA [11], allowing for easy access, navigation and search of all molecular pathways implicated in RA, serving thus, as an on line knowledge base for the disease. The user will have access to all literature used, with detailed annotations for every component and reaction, including PubMed IDs, and a list of identifiers such as Uniprot, EntrezGene, Ensembl, HGNC and RefSeq. As the map is constructed using information from various experimental studies, the user will also be able to opt for visualization of data with specific cell origin, highlighting cell-specific sub-networks within the global one. Moreover, the user will have the possibility to spot all known drug targets, and the corresponding drugs up to date for RA. Detailed view of an element will allow the search for drugs, chemicals and miRNAs targeting this particular element. Additionally, user-provided omic datasets could be displayed as overlay, giving a first estimation of affected pathways and components. Lastly, the map will provide feedback about the unmapped molecules from the dataset, allowing for better understanding of the experimental results and for further development of the map’s contents. We have used public datasets from proteomic and transcriptomic studies [12–14] to demonstrate how the map can be used as a template for separate or simultaneous visualization of different experimental results. The map will also be used for the mapping of in-house data concerning the transcriptome analysis of ten individuals that developed RA (measurements before the onset of the disease and early after) (Teixeira *et al*., under preparation).

The RA map so far includes information derived from more than 100 scientific papers. It has six distinct compartments, namely extracellular space (with extracellular proteins), plasma membrane (with membrane receptors and ligand proteins), cytoplasm (with proteins, miRNAs, small molecules and the sub-compartments of mitochondrion, Golgi apparatus and endoplasmic reticulum), nucleus (with genes, RNAs and transcription factors), a compartment for the secreted molecules and a phenotype compartment including more than ten cellular fates. It comprises more than 400 components and a total of 324 reactions. Each component and reaction in the map is referenced with at least two PubMed IDs or database identifiers if inferred from a specific database.

Topological analysis of the RA map using the software Cytoscape [15] and relevant plugins reveals unconnected or loosely connected parts that reflect our fragmented knowledge about physical and/or genetic interactions, posing thus obstacles in the subsequent derivation of a reliable dynamical model. To improve connectivity we use dedicated PPI databases (through http://www.imexconsortium.org), pathway databases (e.g. KEGG, SIGNOR or REACTOME) and the commercial software Ingenuity Pathway Analysis (IPA, http://www.ingenuity.com) in order to investigate potential co-players of the proteins of interest. For the time being, we do not make use of simulated/computationally inferred interactions or interactions inferred from other species (i.e. mice), restricting our search to experimentally validated data of human origin.

Characteristic features of RA include synovial inflammation that can lead to bone erosion and permanent deformity. It is broadly recognized that in RA, synovial inflammation results from complex interactions between haematopoietic and stromal cells. Recent studies have shown that RA synovial fibroblasts play a crucial role in driving the persistent, destructive characteristics of the disease [16].

The second scope of the project is to model synovial fibroblasts behavior under different initial conditions specific to RA, in order to see if we could influence the cellular fate (e.g. enhancing an apoptotic phenotype) or understand what could lead to patient’s resistance to a certain drug and how to overcome it (e.g. presence of rescue pathways, complex feedback mechanisms).

In general, pathway representation and modelling can be seen as two separate tasks with different primary objectives. The first is to draw an accurate, comprehensive diagram depicting current biological knowledge while the second is to study the emergent behavior of the system under different conditions. However, a detailed, fully annotated molecular map works as an excellent scaffold for the building of a regulatory graph and the subsequent derivation of the logical model. This process, that involves many iterations, obliges one to look meticulously into the mapped pathways, spotting potentially problematic or ambiguous aspects of the map. Model simulations can also reveal inconsistencies concerning the global behavior, advocating the necessity for further revisions and refinements. Leaning on the RA knowledge base and using the web platform Cell Collective [17], we are currently building a Boolean dynamical model for the study of RA fibroblasts’ activation. The model is based on a previously published, more generic model on fibroblasts [18] that is being modified accordingly in order to be RA specific.

In Boolean formalism, nodes represent regulatory components (proteins, complexes, transcription factors, etc.) and arcs represent their interactions. Each regulatory component is associated with a Boolean variable (taking the values 0 or 1) denoting its qualitative concentration or level of activity (0 for absent or inactive, 1 for present or active). The future state of each node depends on the states of its upstream regulators and is defined by a Boolean function, expressed in the form of a rule using the logical operators AND, OR and NOT.

The tuning of the model includes testing against published data and appropriate modifications of logical rules and/or addition/deletion of interactions/components until it is able to reproduce well established input-output relations (global behavior). This process will inevitably lead back to the re-evaluation of the molecular map and further discussions with experts until all issues are resolved in a biologically sound way. The model will then be used to systematically test different initial conditions and stimuli (presence or absence of different cytokines and growth factors, and their combinations). The aim is to predict the system’s response to single or combined perturbations, and identify novel targets for pharmacological intervention.

The Boolean model will be made publicly available in Cell Collective for further contributions, simulations, and analyses by the research community, hopefully within 2018. The web based platform Cell Collective allows real time simulations without the need for software installation making the model more accessible to a wider audience. Moreover, the platform supports annotation, so the user can have simultaneous access to the model description, the rules and the literature used for the rules’ inference.

Lastly, the resulting logical model for RA fibroblasts could be further analyzed with the software GINsim [19] and also serve as a template for the derivation of a continuous model using the software MaBoSS [20] allowing the computation of phenotype probabilities.

## Figures and Tables

**Figure 1:**
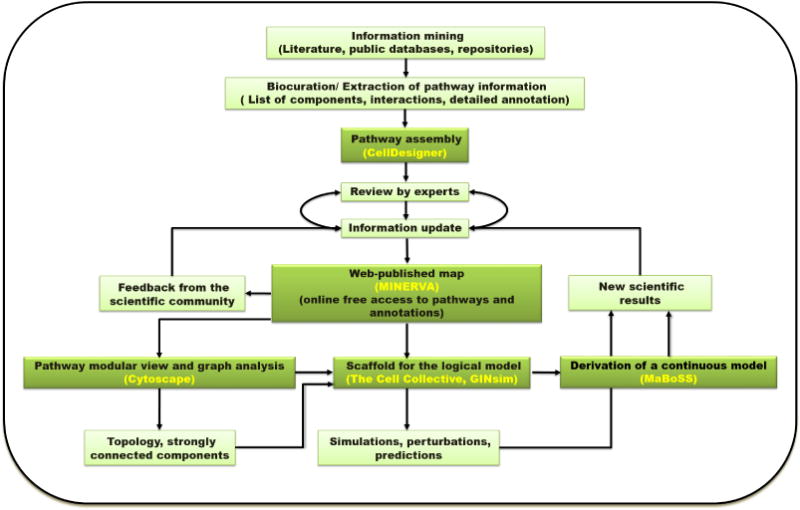
Data integration workflow The building of a logical model is an iterative multistep process. The assembly of a molecular map comprising biological pathways of interest and integrating information from literature and public databases could serve as the first step. Experts’ feedback assures the quality of the map and the accuracy of the knowledge represented, along with strict curation criteria and standards for the graphical representation. Web publication facilitates community feedback and transforms the map in a powerful data analysis and visualization tool. The network can be further exploited using graph analysis tools to identify important nodes and pathways, or it can serve as a scaffold for dynamical models allowing simulations. Interesting predictions can then be experimentally tested, contributing to the validation and refinement of the map. Regular revisions are also necessary to ensure the incorporation of novel data (Figure adapted from Niarakis *et al.*, 2014 [3]).
